# **Real-time PCR applications for diagnosis of leishmaniasis**

**DOI:** 10.1186/s13071-018-2859-8

**Published:** 2018-05-02

**Authors:** Luca Galluzzi, Marcello Ceccarelli, Aurora Diotallevi, Michele Menotta, Mauro Magnani

**Affiliations:** Department of Biomolecular Sciences, University of Urbino Carlo Bo, via Saffi 2, 61029 Urbino, PU Italy

**Keywords:** HRM, *Leishmania*, Leishmaniasis, Melting curve analysis, Molecular diagnosis, qPCR, Real-time PCR

## Abstract

Leishmaniasis is a vector-borne disease caused by many *Leishmania* species, which can infect both humans and other mammals. Leishmaniasis is a complex disease, with heterogeneous clinical manifestations ranging from asymptomatic infections to lesions at cutaneous sites (cutaneous leishmaniasis), mucosal sites (mucocutaneous leishmaniasis) or in visceral organs (visceral leishmaniasis), depending on the species and host characteristics. Often, symptoms are inconclusive and leishmaniasis can be confused with other co-endemic diseases. Moreover, co-infections (mainly with HIV in humans) can produce atypical clinical presentations. A correct diagnosis is crucial to apply the appropriate treatment and the use of molecular techniques in diagnosis of leishmaniasis has become increasingly relevant due to their remarkable sensitivity, specificity and possible application to a variety of clinical samples. Among them, real-time PCR (qPCR)-based approaches have become increasingly popular in the last years not only for detection and quantification of *Leishmania* species but also for species identification. However, despite qPCR-based methods having proven to be very effective in the diagnosis of leishmaniasis, a standardized method does not exist. This review summarizes the qPCR-based methods in the diagnosis of leishmaniasis focusing on the recent developments and applications in this field.

## Background

Leishmaniasis is caused by protozoan parasites of the genus *Leishmania*. This genus includes three subgenera: *Leishmania*, *Viannia* and *Sauroleishmania*. Each subgenus presents different complexes and each complex includes several species [1]. Depending on the *Leishmania* species and host characteristics, the infection can be asymptomatic or it can lead to a spectrum of diseases, notably cutaneous leishmaniasis (CL), visceral leishmaniasis (VL) or mucocutaneous leishmaniasis (MCL). About 12 million people are affected by the disease; moreover, 0.2–0.4 million cases per year and 0.7–1.2 million cases per year have been estimated for VL and for CL, respectively [2]. Infections are widespread both in the Americas (New World) and in Europe, Africa, Asia (Old World), therefore constituting an important global health problem.

The diagnosis of leishmaniasis relies on clinical manifestations, epidemiological and laboratory data. Concerning the laboratory methods, a gold-standard for human patients or animals is lacking [3], impairing accurate epidemiological data collection and thus limiting the disease control. Moreover, false-negative results could delay treatment, thus contributing to reservoirs maintenance. Several immunological and molecular diagnostic tools for diagnosis of leishmaniasis have been developed recently [4]. In particular, the use of molecular techniques has become increasingly relevant due to their high sensitivity, specificity and possible application to a variety of clinical samples. Among them, the real-time PCR, also named quantitative PCR (qPCR), has become increasingly popular recently since it is fast, has broad dynamic range, and cross-contamination is drastically reduced because there is no need to open reaction tubes for post-PCR analyses. The qPCR relies on analysis of fluorescent signal produced during amplification. Fluorescence can be generated by using intercalating fluorescent dyes (e.g. SYBR Green) or fluorescent probes (e.g. TaqMan®). The assays based on intercalating dyes are characterized by high sensitivity, as long as primers are highly specific to the target sequence to avoid generation of non-specific products that would lead to overestimated or false positive results. A melting curve analysis can be performed post-PCR to ensure the presence of a single specific amplicon. For genotyping purposes, high resolution melt (HRM) analysis can be used to differentiate amplicons based on sequence variations [5]. The use of probes is typically more expensive. However, using probes allows multiplexing (partially reducing the cost per assay) and furnish additional specificity to the assay. Therefore, this approach is less subject to false positives than the intercalating dye method.

A search in PubMed in February 2018 with the wildcard term “Leishmania*” in conjunction with the search terms “real-time PCR or qPCR”, found over 540 published manuscripts from 2001 to February 2018. A further combination with the wildcard terms “quantif*” or “detect*” or “diagnos*” and a manual sift of the papers identified nearly 180 manuscripts focusing on the topic of this review. Nevertheless, many of these publications apply similar assays.

The pioneering works of Bretagne et al. [6] and Nicolas et al. [7] described two qPCR-based methods to detect and quantify *Leishmania* parasites using the DNA polymerase gene and the minicircles kinetoplast DNA (kDNA) as a target, respectively. Since then, a variety of qPCR-based assays have been developed on different molecular targets, not only for detection and quantification of *Leishmania* species but also for genotyping and species identification. Regarding the detection chemistry, SYBR Green and TaqMan® probes were most widely employed, followed by other probes, such as FRET probes [8, 9] or MGB probes [10]. The sensitivity and specificity reported for published qPCR assays is variable. For instance, qPCR assays developed for VL diagnosis in humans showed a specificity variation between 29.6–100% and sensitivity between 91.3–100%, indicating the usefulness of the qPCR when a sensitive tool is pivotal [4].

In this review, recent developments and applications of qPCR-based methods in the diagnosis of leishmaniasis are summarized, highlighting advances and limits of this powerful technique.

## Molecular targets

Many qPCR-based approaches for the diagnosis of leishmaniasis have been published based on coding and/or non-coding regions in the *Leishmania* genome. *Leishmania* spp. have 34–36 chromosomes and a unique genomic organization in which protein-coding genes are organized in polycistronic units and do not have introns; moreover, gene expression is regulated post-transcriptionally (i.e. at the levels of mRNA stability and translation) [11]. *Leishmania* parasites also possess a mitochondrial genome called kinetoplast DNA, which is organized in thousands of minicircles (0.8–1.0 Kb each) and several dozens of maxicircles (approximately 23 Kb each) linked in a concatenated network.

### Non-protein-coding regions

The kDNA minicircles account for approximately 95% of kDNA and encode small guide RNAs (gRNAs), needed for RNA-editing of the transcripts encoded by maxicircles [12]. Since minicircles are present in thousands of copies per cell, they are ideal targets for highly sensitive detection of *Leishmania* [13, 14]. Each minicircle is composed of a conserved region containing the origin of replication, and a variable region encoding usually a single gRNA [15]. The minicircle network is composed of different minicircle classes. Their number can be variable and dependent from strain; in a recent work, the presence of over 100 minicircles classes has been demonstrated in a strain of *L. tarentolae* using NGS technology [16].

The minicircle conserved region contains 3 conserved sequence blocks (CSB-1, CSB-2 and CSB-3) that could be effective targets for PCR amplification of all minicircle classes. However, polymorphisms have been shown in CSB-1 region of *L. infantum* [14] and, using primers amplifying a subpopulation of minicircles, also in CSB-2 region of New World species [17]. In general, the assays designed on minicircles conserved regions identify *Leishmania* parasites only at the genus or subgenus level. In fact, qPCR assays intended for a single species designed on minicircles can potentially amplify more than one *Leishmania* species, as shown by several authors [14, 18–22].

Among targets on chromosomal DNA, different regions of ribosomal RNA (rRNA) genes, termed ribosomal DNA (rDNA), have been used. Tens or hundreds of copies of rDNA unit can be present per *Leishmania* cell, allowing sufficient sensitivity for analyzing clinical sample DNA [23, 24]. Each rDNA repeat unit consists of several genes and spacers: the *SSU* rRNA gene (18S) is followed by *5.8S* and *LSU* genes; two internal transcribed spacer regions -*ITS1* and *ITS2* - are located between the *18S* and *5.8S* genes and between *5.8S* and *LSU*α genes, respectively [25]. The *18S* rDNA region, due to its highly conserved nature, is commonly utilized to design primers and/or probes for the diagnosis of *Leishmania* spp. [26]. On the other hand, the *ITS* regions, which have more variable sequences, can be used for typing at the species level [24, 27, 28].

In principle, qPCR assays designed on minicircles kDNA conserved regions allow to reach the highest limit of detection, due to their high number per parasite. In fact, a detection limit of 5 × 10^-4^ parasites per PCR reaction tube, with a dynamic range of 10^7^, has been reported for *L. infantum*, allowing to detect up to 0.0125 parasites/ml of blood [18]. In general, the sensitivity limit depends on assay design (which target region is selected), chemistry used (intercalating dyes or fluorescent probes), the nature of clinical sample and the DNA extraction method. For instance, Gomes et al. [22] compared the sensitivity of two qPCR assays (based on SYBR Green or TaqMan® probe) targeting *Leishmania* (*Viannia*) kDNA using swabs and biopsy samples from MCL and CL patients. The authors showed that sensitivity did not vary significantly by sample type, but rather according to the method: the SYBR green-based assay reached the higher level of sensitivity. It is also important to emphasize that the observed higher sensitivity of SYBR green might reflect differences in DNA sequences at primer annealing sites, rather than the qPCR chemistry itself.

### Protein-coding sequences

Many protein coding genes have also been used as target in qPCR assays for identification/quantification of the parasites. Among them are the Heat Shock Protein 70 kDa (*HSP70*) [20, 27, 29, 30], DNA polymerase [6, 20, 31–34], glucose-6-phosphate dehydrogenase (*G6PD*) [13, 35], glucose phosphate isomerase (*GPI*) [36], mannose phosphate isomerase (*MPI*), 6-phosphogluconate dehydrogenase (*6PGD*) [9], tryparedoxin peroxidase [37], etc. These targets have in general high specificity but lower sensitivity compared to targets with high copy numbers, such as kDNA minicircles or rDNA. *HSP70* could be considered an exception, since it is a multicopy gene [38]. In fact, an optimized qPCR assay showed a limit of detection of 0.1 parasites/ml of culture [30]. *HSP70* gene has been widely used for *Leishmania* phylogenetic studies [39] and its sequence heterogeneity has been implemented in several PCR-RFLP approaches [40] and qPCR-based typing methods (see below). The *G6PD*, *MPI*, *GPI* and *6PGD* genes encode for enzymes used in multi-locus enzyme electrophoresis (MLEE) analysis and were also used in multi-locus sequence typing (MLST) approaches [41]. The limit of detection of qPCR assays designed on single copy genes can be high (e.g. 5.6 pg parasite DNA per reaction) [36]. In other cases (using DNA polymerase or *G6PD* as target) the limit of detection has been reported in 0.4 parasites/reaction [32] and 10 target copies/reaction [13].

## Clinical samples

To establish a qPCR-based method for diagnosis of leishmaniasis, other than selection of target sequence (size, genetic stability, copy number, level of specificity) and characterization of assay performance (specificity, sensitivity, accuracy and reproducibility), it is essential to test the assay with clinical samples.

The correct sampling is often critical for success of downstream test [42]; in fact, clinical samples can be heterogeneous, depending on clinical presentation (CL, VL, MCL). For CL, nucleic acids can be extracted from a full-thickness skin biopsy specimen collected from a lesion, while for MCL, biopsy specimens should be collected from mucosal areas with abnormalities [43]. Also swabs from CL or MCL lesions are emerging as a powerful diagnostic tool because of its non-invasive and simple collection method [22, 44]. For VL, bone marrow or lymph-node aspirates, as well as whole blood or buffy coat, are common source of tissue samples [43]. Recently, the detection of *L. infantum* kDNA by qPCR was also demonstrated in urine of untreated individuals affected by VL. The fact that parasite’s DNA was not found after start of therapy indicated that urine could be considered a potential alternative specimen to follow up the efficacy of therapeutic approaches [45]. The presence of *L. infantum* kDNA in urine was also shown in dogs with natural clinical leishmaniasis, although in lower amounts compared to blood or bone marrow [46]. In canine or feline leishmaniasis (CanL or FeL), conjunctival swabs have shown to be a valid alternative to other more invasive clinical samples, such as blood, bone marrow or lymph-node aspirates [34, 47–50]. Also nasal, oral or ear swabs have been successfully tested [33, 51].

Real-time PCR has been used to explore the presence of *Leishmania* DNA in tissues of wild mammals both in New World and Old World, to find potential parasite reservoirs. For example, *Leishmania* DNA was found from rodent and bat spleen tissues [52–55]. Furthermore, *L. infantum* DNA has been also retrieved from hair of wild mammals, such as Leporidae [56], fox, wolf, rat, marten and hedgehog [57].

All clinical samples require the use of an internal reference (generally a host sequence, e.g. β-actin or beta-2-microglobulin), not only to monitor PCR inhibition (i.e. false-negative results), but also to normalize the parasite load to the amount of host cells [33, 34, 48, 58]. Although some clinical samples may contain a small amount of parasite DNA, the use of high copy number sequence as targets (e.g. kDNA minicircle, rDNA) can ensure adequate sensitivity.

## qPCR assays for parasite detection and quantification

As outlined above, numerous quantitative and qualitative real-time PCR-based assays to be used in veterinary or human medicine have been published recently, targeting different genetic markers and for application to different types of samples [51, 59–69]. The main features of qPCR assays targeting kDNA, rDNA or other DNA regions are summarized in Table 1, Table 2 and Table 3, respectively.

**Table 1:** Summary of the characteristics of real-time PCR assays targeting *Leishmania* kDNA, in chronological order

Geographical region	Type of sample	Assay type	Assay chemistry	Typing	Reference
Old World	Mouse tissues	Quantitative	Intercalating dye	No	[7]
Old World	Parasite cultures	Qualitative	Intercalating dye	Yes	[96]
Old World	Blood/ bone marrow	Quantitative	Fluorescent probe	No	[18]
Old World	Blood/ lymph node/skin	Quantitative	Fluorescent probe	No	[65]
Old World	Blood/ lymph node/skin	Quantitative	Fluorescent probe	No	[67]
Old World	Blood/ bone marrow	Quantitative	Fluorescent probe	No	[80]
Old World	Urine/ blood/ bone marrow	Quantitative	Fluorescent probe	No	[46]
Old World	skin lesions	Qualitative	Intercalating dye	Yes	[97]
New World	blood	Quantitative	Intercalating dye	No	[106]
Old World	Blood/ bone marrow	Quantitative	Fluorescent probe	No	[3]
New World	Bone marrow	Qualitative	Intercalating dye/ Fluorescent probe	No	[62]
Old World/ New World	Blood/ skin biopsy	Quantitative	Intercalating dye/ Fluorescent probe	Yes	[20]
New World	Sand flies	Quantitative	Intercalating dye	No	[32]
Old World	Blood	Qualitative	Intercalating dye	No	[70]
Old World	Ticks	Quantitative	Fluorescent probe	No	[94]
Old World	Blood/ lymph node/ Oral swab/ Conjunctival swab	Quantitative	Fluorescent probe	No	[50]
Old World	Blood/ ticks	Quantitative	Fluorescent probe	No	[93]
New World	Blood/ bone marrow/ skin biopsy/ sand flies	Qualitative	Intercalating dye	Yes	[98]
Old World	Blood/ bone marrow	Quantitative	Fluorescent probe	No	[61]
New World	Blood/ skin biopsy	Quantitative	Intercalating dye	Yes	[59]
New World	Skin biopsy	Quantitative	Intercalating dye	No	[13]
Old World	Blood/ lymph nodes/ bone marrow/ conjunctival swab	Qualitative	Intercalating dye	No	[64]
Old World	Blood/ lymph node/ dog hairs	Quantitative	Fluorescent probe	No	[63]
New World	Blood /bone marrow/ skin biopsy/ conjunctival swab	Qualitative	Intercalating dye	No	[49]
Old World/ New World	Blood/ conjunctival swab	Quantitative	Intercalating dye	Yes	[14, 48]
New World	Sand flies	Qualitative	Intercalating dye	No	[88]
New World	Skin biopsy/ scraping/ cytology brushes	Quantitative	Intercalating dye	No	[78]
Old World/ New World	Liver	Quantitative	Fluorescent probe	No	[19]
New World	Oral swabs/ conjunctival swab/ blood/ lymph node	Quantitative	Intercalating dye	No	[51]
New World	Blood	Qualitative	Intercalating dye	Yes	[99]
New World	Skin	Quantitative	Intercalating dye	No	[107]
New World	Blood/ urine	Quantitative	Intercalating dye	No	[45]
New World	Lesion swab/ biopsy	Qualitative	Intercalating dye/ Fluorescent probe	Yes	[22]
Old World	Sand flies	Qualitative	Intercalating dye	No	[92]
Old World/ New World	Blood/ conjunctival swab	Quantitative	Intercalating dye	Yes	[17]
Old World	Sand flies	Quantitative	Intercalating dye	No	[87]
New World	Parasite cultures	Quantitative	Intercalating dye	No	[30]
New World	Blood	Quantitative	Fluorescent probe	No	[81]
New World	Blood/ splenic aspirate/skin	Quantitative	Fluorescent probe	No	[82]
New World	Cerebrospinal fluid/ CNS tissues/ spleen	Quantitative	Fluorescent probe	No	[108]
New World	Lymph node/ bone marrow	Quantitative	Intercalating dye	Specific for *L. infantum*	[109]

**Table 2:** Summary of the characteristics of real-time PCR assays targeting *Leishmania* rDNA, in chronological order

Geographical region	Type of sample	Assay type	Assay chemistry	Typing	Reference
Old World	Blood	Quantitative	Fluorescent probe	No	[26]
Old World/ New World	Blood/ bone marrow/ skin biopsy	Quantitative	Fluorescent probe	Yes	[101]
New World	Cell cultures	Quantitative	Intercalating dye	No	[110]
New World	Skin biopsy	Quantitative	Fluorescent probe	No	[111]
Old World	Mouse skin biopsy	Quantitative	Fluorescent probe	No	[112]
Old World	Bone marrow	Quantitative	Intercalating dye	No	[113]
Old World	Smears from cutaneous lesions/ blood/ skin or spleen biopsy	Quantitative	Intercalating dye	Yes	[28]
New World	Sand flies	Quantitative	Intercalating dye	No	[32]
New World	Blood/skin biopsy	Quantitative	Intercalating dye	Yes	[59]
Old World	Blood/ bone marrow/ lymph node/cutaneous lesion aspirates	Qualitative	Fluorescent probe	Yes	[71]
Old World	Fixed lymph node biopsy	Quantitative	Fluorescent probe	Specific for *L. tropica*	[114]
New World	Swab/ lesion aspirates	Quantitative	Fluorescent probe	No	[44]
New World	Parasite cultures	Qualitative	Intercalating dye	Yes	[27]
Old World	Blood/ bone marrow	Quantitative	Intercalating dye	Yes	[102]
Old World	Fixed skin biopsy	Quantitative	Fluorescent probe	No	[115]
New World	Ticks	Qualitative	Intercalating dye	No	[95]
Old World/ New World	Blood/ skin biopsy/ bone marrow	Qualitative	Intercalating dye	Yes	[58]
Old World	Sand flies	Qualitative	Fluorescent probe	Yes	[92]
New World	Skin biopsy	Quantitative	Intercalating dye	No	[76]
New World	Parasite cultures	Quantitative	Intercalating dye	No	[30]

**Table 3:** Summary of the characteristics of real-time PCR assays targeting *Leishmania* sequences other than kDNA or rDNA, in chronological order

Target sequence	Geographical region	Type of sample	Assay type	Assay chemistry	Typing	Reference
DNA pol	Old World	Liver biopsy	Quantitative	Fluorescent probe	No	[6]
*GPI*	Old World/ New World	Skin biopsy	Qualitative	Fluorescent probe	Yes	[36]
DNA pol	New World	Sand flies	Quantitative	Fluorescent probe	No	[85]
*GP63*	Old World	Mice tissues	Quantitative	Intercalating dye	Yes	[68]
*G6PD*	New World	Biopsy	Quantitative	Intercalating dye/ Fluorescent probe	Yes	[35]
DNA pol	New World	Sand flies	Quantitative	Intercalating dye	No	[32]
Alpha-tubulin, DNA pol, Spliced leader, MSP-associated gene (MAG), SIDER repeat, *GPI*, *HSP70*, splice leader-associated retrotransposons (SLACS)	Old World/ New World	Blood/ skin biopsy	Quantitative	Intercalating dye/ Fluorescent probe	Yes	[20]
tryparedoxin peroxidase	Old World	Skin biopsy	Qualitative	Intercalating dye	Yes	[37]
DNA pol	New World	Skin biopsy/ bone marrow/ blood/ conjunctival swab	Quantitative	Fluorescent probe	No	[34]
DNA pol	New World	Bone marrow/ skin biopsy/ nasal, oral, ear swab	Quantitative	Fluorescent probe	No	[33]
*MPI*, *6PGD*	New World	Scrapings/ skin biopsy	Qualitative	Fluorescent probe	Yes	[9]
*G6PD*	New World	Biopsy	Quantitative	Intercalating dye	No	[13]
*HSP70*	New World	Parasite cultures	Qualitative	Intercalating dye	Yes	[27]
*AAP3*	Old World/ New World	Mouse tissues	Quantitative	Fluorescent probe	No	[116]
REPL repeats (L42486.1)	Old World	Blood	Quantitative	Fluorescent probe	Specific for *L. infantum* and *L. donovani*	[117]
LinJ31	Old World/ New World	Lymph node/ bone marrow aspirate/ tissue samples	Quantitative	Fluorescent probe	Specific for subgenus *Leishmania*	[118]
*CPB*	Old World	Cutaneous biopsy	Qualitative	Fluorescent probe	Yes	[8]
*HSP70*	Old World/ New World	Biopsy/ sand flies	Qualitative	Intercalating dye	Yes	[29]
IPPP, RNase III	New World	Sand flies	Quantitative	Intercalating dye	Yes	[119]
REPL repeats (L42486.1)	Old World	Blood/ skin	Quantitative	Fluorescent probe	Specific for *L. infantum* and *L. donovani*	[69]
*HSP70*	New World	Parasite cultures	Quantitative	Intercalating dye	No	[30]

Several assays developed for the diagnosis of leishmaniasis report only qualitative results (i.e. positive or negative detection) [22, 70]. These qualitative assays exploit the advantages of real-time technology to reduce the time of analysis and the risk of contamination. Moreover, many of these assays were often finalized to obtain genotyping information (see below) [8, 71].

Other assays were developed to estimate the parasite load through absolute or relative quantification. The absolute quantification relies on the use of a standard curve, obtained with serial dilutions of purified parasite genomic DNA or target sequence. The use of high-copy *18S* and minicircle kDNA sequences as a target usually allows amplification at the genus or subgenus level, due to their conserved sequences or to the heterogeneity of minicircle classes [72]. The reliability of quantification results is dependent on (i) the knowledge of the target sequence copy number/cell, and (ii) the fact that this number does not vary significantly among species or between the reference and field strains. Concerning minicircle kDNA, their copy number can vary between *L.* (*Leishmania*) species or between isolates within a single species [18, 20], potentially affecting the quantification accuracy of qPCR assays. However, no statistically significant differences in the relative number of kDNA minicircle targets among three *L.* (*Viannia*) species were found [13]. Moreover, the minicircle kDNA copy number within the same strain or in amastigotes infecting a single patient during a survey appears stable; therefore, a qPCR assay designed on this target can be used to compare parasitemia levels during the survey of the patient, and can be particularly useful for relapse monitoring [18]. Changes in minicircle copy numbers during stage transition (promastigotes-amastigotes) has also been investigated in a *L. chagasi* strain (*L. chagasi* has been shown to be identical to *L. infantum* from southwest Europe [73]). No significant differences were found, concluding that standard curves generated from promastigote-derived DNA can be used for quantification of amastigotes of the same species/strain [20].

It is also noteworthy that minicircle sequence heterogeneity could be lost during continuous culturing [74]; also, it has been shown that *in vitro* selection of drug-resistant *L. amazonensis* lead to changes in kDNA minicircle class dominance [75]. Single-copy targets do not present these drawbacks and could lead to better quantitative accuracy, but their sensitivities can be too low for diagnostic purposes using samples with low parasite loads and without parasite isolation/cultivation. For example, the single copy gene DNA polymerase I has been used to normalize minicircle kDNA copy numbers using the comparative quantification (ΔΔCt) method [18, 20].

When using clinical samples, the presence of host DNA as background should be taken into account. In fact, excess of background DNA could affect the reliability of qPCR results. Therefore, standard curves should be tested by adding appropriate amounts of host DNA into PCR mixtures to mimic amplification in clinical samples. In some cases, the presence of background DNA may affect Ct values: delays of several cycles were observed in qPCR assays targeting minicircle kDNA using *Leishmania* DNA samples spiked with human or canine DNA (100 ng and 30 ng, respectively) [14, 17]. Nevertheless, the linearity range of qPCR remained unchanged up to the assay detection limits. In other cases, the background host DNA did not appear to affect the Ct values. For example, in a qPCR targeting *G6PD*, similar Ct values were obtained in the presence *versus* absence of human DNA (20 ng) and sensitivity was not affected by the presence of this background DNA [13]. We also tested the use of conjunctival swabs (CS) for the evaluation of CanL by qPCR targeting kDNA. Since we used raw CS lysates as source of *Leishmania* DNA, their potential inhibition in qPCR was tested: it was found that 1 μl raw lysate per 25 μl qPCR reaction mixture did not show any inhibitory effect on the assays, allowing accurate quantification [48].

While using blood as clinical sample gives results as parasites/ml, the quantification on solid samples (swabs, biopsies, etc.) requires the quantification of both parasites and the host cells (by means of another qPCR for a single copy host gene). The comparison of the two qPCR results allows to obtain the number of parasites/number of host cells [13, 33, 34, 48, 76, 77]. Alternatively, if yield of DNA extraction is reproducible among different samples and the amount of DNA can be accurately determined, the number of parasites could also be normalized to μg of tissue DNA [78]. In addition to the parasite and host cell quantification, a ΔCt value between amplification curves of the *Leishmania* target and a host reference gene (Ct *Leishmania -* Ct host gene) was used as an additional parameter to monitor parasite load changes during the course of the illness or treatment. In fact, increases or decreases in ΔCt values correlate with decreases or increases in parasite loads, respectively [48, 79]. This comparison is possible only if qPCR efficiencies of *Leishmania* and host gene are similar. This approach, based on ΔCt calculation of two qPCR assays, could get around the problem of exact parasite load quantification (based on standard reference strains) and could avoid the use of two standard curves, therefore limiting the cost of assays [48].

Recently, efforts have been made to reduce cost and time necessary to obtain diagnostic results, as well as to decrease manipulation steps, therefore reducing the risk of mistakes or contamination. For example, a real-time PCR assay previously developed by Francino et al. [80] for the detection and quantification of *L. infantum* kinetoplast DNA, has been optimized in terms of lower reaction volume and shorter running time [81]. Moreover, Rampazzo et al. [82] developed a ready-to-use (gelified and freezer-free) duplex qPCR for the identification of *L. infantum* in dogs. In this assay, a qPCR for detection of the canine *18S* rRNA gene and a qPCR for *L. infantum* kDNA detection were combined, showing a detection limit of 1 parasite/reaction in all canine tissues tested (splenic aspirates, skin and blood).

The qPCR assays for leishmaniasis that amplify species-specific DNA sequences are not appropriate for regions with sympatric *Leishmania* species [83]. In fact, even in areas where *Leishmania* species are known, the presence of polymorphisms and variations in copy number of target sequences (e.g. minicircle classes), could interfere with the performance of qPCR assays. To rule out false negatives or co-infections with other *Leishmania* species, the species-specific qPCR should be combined with a genus-specific assay (broad range assay) [24]. Therefore, approaches based on initial amplification of genus-specific sequences followed by assays for species differentiation/typing should be useful.

## qPCR assays for *Leishmania* monitoring in insect vectors

The arthropod vectors involved in the *Leishmania* transmission are only the phlebotomine sand flies, with 98 species of the genera *Phlebotomus* and *Lutzomyia* proven or suspected to be vectors of human leishmaniasis [84]. Since microscopic detection of parasites in sand fly guts is laborious and time-consuming, biomolecular assays were introduced with the aim to investigate the potential role of sand fly species in spreading *Leishmania* parasites. In 2008 Ranasinghe et al. [85] proposed a qPCR assay to detect *L. chagasi* DNA in *Lutzomyia longipalpis*, using the DNA polymerase gene of parasite as molecular target and a TaqMan® probe, with a detection limits of 10 pg *Leishmania* DNA (∼120 parasites) in 100 ng sand fly DNA. The quantification of parasite load in the vector can be very important. In fact, it is well known that *Leishmania* parasites can manipulate sand fly feeding behavior since a high parasite load in sand fly midguts is correlated to a persistent feeding pattern that leads to an increase in *Leishmania* transmission [86]. The principal mechanism proposed is the blockage of the stomodeal valve that forces sand flies to remain feeding for longer on the same or different hosts, obtaining often only a partial blood meal. This fact can increase the likelihood of parasite transmission [87]. Thus, considering the importance of a quantitative test, Bezerra-Vasconcelos et al. investigated other molecular targets, with the aim of to improve the assay sensitivity [32]. In their work, the authors analyzed the sensitivity of kDNA, DNA polymerase α and 18S rRNA gene in SYBR-Green-based qPCR assays. The amplification of kDNA exhibited the greatest sensitivity, showing the capacity to detect DNA equivalent to 0.004 parasites, and the highest accuracy. Successively, Cunha et al. [88] investigated the use of kDNA qPCR *versus* kDNA conventional PCR for detection of *L. infantum* DNA in *Lutzomyia longipalpis*, concluding that kDNA qPCR can be used for epidemiological studies since this assay was particularly advantageous when analyzing samples containing a small number of parasites. In a similar approach, a kDNA SYBR-Green qPCR was used to study the outbreak in southwestern Madrid region. The use of a quantitative approach permitted the study of the parasite load in unfed and blood-fed *Phlebotomus perniciosus* female sand flies caught in the focus area. The data obtained allowed the authors to confirm that the high parasite load in females of *P. perniciosus* without blood in their guts is undeniable proof of a proper establishment and replication of the parasites in the sand fly midgut [87]. Using qPCR-based approaches, *Leishmania* DNA has also been detected in sand flies from Albania [89], Tunisia [90] and Turkey [91]. Recently, an interesting approach based on sequential qPCR targeting kDNA followed by *ITS1* qPCR was proposed by Karaku et al. [92] to study a leishmaniasis focus in southwestern Turkey. In fact, in this region different *Leishmania* species coexist, therefore a genus-specific kDNA SYBR-Green qPCR was used to screen for infection in the collected sandflies and qPCR targeting *ITS1* region was performed using species-specific primers for detecting *L. donovani/infantum* complex, *L. tropica* and *L. major*. Moreover, the DNA extracted from the abdomens of freshly blood-fed female sandflies was amplified using the *cytochrome b* gene as a target to identify the vertebrate source of the blood meal and to reveal the host preferences in the study area [92]. Interestingly, the qPCR approach was utilized also to detect *L. infantum* DNA in ticks (*Rhipicephalus sanguineus*) removed from dogs living in endemic areas in Italy, finding the parasite DNA in a fraction of ticks examined [93, 94]. Successively, *Leishmania* spp. promastigotes were detected in the intestine, ovary and salivary glands of *R. sanguineus* actively infesting dogs in Brazil [95]. In the light of these evidences, additional studies will be needed to further explore the role of the ticks in *Leishmania* infection.

## qPCR assays for *Leishmania* genotyping

Both qualitative and quantitative real-time PCR assays can be designed to gain information regarding parasite typing at the subgenus, complex, or species level. Different targets have been evaluated for this purpose, such as *ITS* region, SSU, *HSP70*, *G6PD*, *6PGD*, *MPI*, cysteine protease B (*cpB*), kDNA minicircle, etc. [83]. Various qPCR-based typing methods have been published in recent years. For example, Wortmann et al. developed real-time PCR assays for the detection and differentiation of four *Leishmania* complexes (*L. mexicana*, *L. donovani/infantum*, *L. major* and species belonging to the subgenus *Viannia*). The primers/probes were designed on the glucose phosphate isomerase (*GPI*) gene exploiting the sequence variability of this gene among *Leishmania* species. The limit of detection for all assays was 5.6 pg, equivalent to approximately 165 genome copies [36].

PCR primers that specifically amplify a species or a group of species have been designed on minicircle kDNA, exploiting sequence polymorphisms. However, several authors observed cross-reaction among species, probably because of minicircle classes variability in each of them [17, 19, 20, 23]. In a different approach, using common primers to amplify different species, differences in melting temperature (Tm) of PCR-amplified minicircle kDNA regions were exploited to differentiate *Leishmania* species. Nicolas et al. optimized an assay to distinguish the Old World species *L. major*, *L. donovani*, *L. tropica* and *L. infantum*. However, this assay could not differentiate *L. tropica* and *L. infantum* by their Tm values [96]. Successively, De Monbrison et al. [97] used the same primers in a real-time PCR followed by melting curve analysis to monitor *Leishmania* species in CL patients from Algeria, confirming prevalence of *L. major* (44.1%). However, about 30% of clinical samples were not determinable. In another study, the Tm analysis of amplicons generated from the conserved region of minicircle kDNA allowed the differentiation of the subgenus *L.* (*Leishmania*) from *L.* (*Viannia*), but species identification was not possible [98]. Successively, de Morais et al. [99] identified two Tm ranges using previously developed primers, designed on minicircle kDNA [59], to differentiate two groups of species causing CL in Brazil. The group 1 included *L.* (*V.*) *braziliensis*, *L.* (*V.*) *panamensis*, *L.* (*V.*) *lainsoni*, *L.* (*V.*) *guyanensis*, *L.* (*V.*) *shawi* (Tm = 78–79.99 °C), and the group 2 included *L.* (*V.*) *naiffi*, *L.* (*L.*) *amazonensis* and *L.* (*L.*) *mexicana* (Tm = 80–82.2 °C). A total of 223 positive blood samples were analyzed by qPCR followed by Tm analysis, and the results were compared with other techniques such as SSU sequencing, kDNA PCR-RFLP and MLEE. No significant differences were found between qPCR and MLEE or SSU sequencing methods, while a highly significant difference was observed for qPCR and RFLP.

Approaches based on Tm analysis were also attempted using tryparedoxin peroxidase gene [37]. In this case, a SYBR-green based qPCR on tryparedoxin peroxidase gene incorporating a melting step analysis was developed to discriminate between *L.* (*L.*) *major* and *L.* (*L.*) *tropica* causing cutaneous leishmaniasis in Iran [37].

Melting analysis was also performed using fluorescence resonance energy transfer (FRET) probes [100]. First to apply this technology for *Leishmania* typing, Schulz et al. designed a qPCR assay with FRET probes using SSU region as target [101]. The assay showed analytical sensitivity of 94.1 parasites/ml blood (CI 95%, 70–145.3 parasites/ml) and melting probes analysis distinguished three clinically relevant groups (*L. donovani* complex, *L. braziliensis* complex, and other *Leishmania* spp.). More recently, Tsukayama et al. [9] exploited the polymorphisms in the genes *MPI* and *6PGD* to design two real-time PCR assays based on FRET technology and melting curve analysis. These assays identified five *Leishmania* species of the *Viannia* subgenus highly prevalent in South America: *L.* (*V.*) *braziliensis*, *L.* (*V.*) *panamensis*, *L.* (*V.*) *guyanensis*, *L.* (*V.*) *peruviana* and *L.* (*V*.) *lainsoni*. However, to increase sensitivity of the assays with clinical samples, conventional PCRs for *MPI* and *6PGD* genes were carried out prior to the nested real-time PCR amplifications, but this impaired the possibility of quantification. An approach based on FRET technology and melting curve analysis was also used by Nath-Chowdhury et al. [8] to differentiate Old World CL species [*L.* (*L.*) *major*, *L.* (*L.*) *tropica*, and *L.* (*L.*) *aethiopica*], using the *cpB* gene as target. Moreover, Toz et al. [71] developed a real-time PCR assay on *ITS1* to identify the Old World species *L.* (*L.*) *tropica*, *L.* (*L.*) *major* and *L.* (*L.*) *donovani* complex, using FRET probes to diagnose and simultaneously differentiate among Turkish species in clinical samples. This assay provided sufficient sensitivity for fast and correct detection of parasites directly from clinical materials, allowing the identification of *L.* (*L.*) *tropica* and *L.* (*L.*) *infantum* as causative agents of human CL, VL and CanL in Turkey [71].

The introduction of high-resolution melt (HRM) technology allowed improved technical approaches for *Leishmania* species identification. The HRM analysis of an amplicon from *ITS1* region was used to identify Old World *Leishmania* species [28]. When tested on 300 samples from human cases, reservoir hosts and sand flies, this approach distinguished *L. major*, *L. tropica*, *L. aethiopica* and *L. infantum/donovani* species. More recently, Cardoso et al. [102] used this assay to evaluate the epidemiological role of red foxes as reservoirs in Portugal. HRM analysis of amplicon from *HSP70* gene allowed the discrimination of eight *Leishmania* New World species, i.e. *L.* (*L*.) *chagasi*, *L*. (*L.*) *amazonensis*, *L.* (*L.*) *mexicana*, *L.* (*V.*) *lainsoni*, *L.* (*V.*) *braziliensis*, *L.* (*V.*) *guyanensis*, *L.* (*V*.) *naiffi* and *L.* (*V.*) *shawi*, and three Old World species, i.e. *L.* (*L.*) *tropica*, *L.* (*L.*) *donovani* and *L.* (*L.*) *major* [29]. This method demonstrated high sensitivity, detecting less than one parasite per reaction, even in the presence of host DNA. In another approach, the sequential amplification and HRM analysis of *HSP70* and then *ITS1*, allowed to discriminate six New World *Leishmania* species (*L. mexicana*, *L. infantum chagasi*, *L. amazonensis*, *L. panamensis*, *L. guyanensis* and *L. braziliensis*) with a detection limit of 10 parasites/ml [27]. HRM analysis has also been applied to minicircle kDNA qPCR, allowing discrimination between subgenera *Leishmania* and *Viannia*, and from *L.* (*L.*) *infantum* and *L.* (*L.*) *amazonensis* reference strains [14]. However, HRM analysis performed on 62 clinical specimens from dogs infected by *L.* (*L.*) *infantum* showed that discrimination between *L.* (*L.*) *amazonensis* and *L.* (*L.*) *infantum* was not possible in 53.4% of cases due to melting peak overlapping or late amplification (Ct ˃ 30) [17]. Therefore, a combination of two qPCR assays was used to distinguish *L.* (*L.*) *infantum* and *L.* (*L.*) *amazonensis*, exploiting the major abundance of a minicircle kDNA class in *L.* (*L.*) *amazonensis* rather than targeting a hypothetical species-specific sequence [17]. Importantly, in a systematic study, Weirather et al. [20] developed a serial qPCR strategy to identify and rapidly differentiate *Leishmania* species and quantify parasites. First, selected screening primers were used to recognize kinetoplast minicircle DNA of all *Leishmania* species. Then, further qPCRs individualized for geographical regions were employed for species identification, combining primers, probes and melt curve analysis. The combination of assays allowed detection, quantification and species determination of *Leishmania* parasites in different samples from patients from Bangladesh or Brazil, with different forms of leishmaniasis [20].

Taken together, real-time PCR based assays have demonstrated reliability and affordability in typing *Leishmania* spp. However, due to parasite variability/heterogeneity, a single method to quantify parasites and determine the species in clinical specimens cannot be established and the need of combining different assays or adapt them to geographical regions has emerged.

## Conclusions

The application of qPCR to the diagnosis of leishmaniasis has contributed to the development of sensitive and specific approaches which are important part of the diagnostic process and allow implementation of early and adequate treatment [103, 104]. Real-time PCR applications for *Leishmania* species detection/quantification and typing represent an advance to classic methodologies in terms of automation and high throughput possibility, rapidity and high sensitivity. Nevertheless, a standardized assay to simultaneously estimate parasite load and genotype the species of interest in a particular geographical area is still lacking. In fact, no consensus exists concerning clinical sample harvesting procedures, DNA extraction methods, target sequences, use of host target as reference for normalization or as quality control, etc. Furthermore, due to heterogeneity of published qPCR-based assays and the fact that each assay has pros and cons, it is difficult to identify an ideal approach. Therefore, a combination of different assays can be proposed for maximizing sensitivity and specificity. Since the first goal of a clinical or epidemiological investigation is to determine the presence or absence of *Leishmania* parasites, a high-copy target ensuring low limit of detection, such as minicircle kDNA, appears the best choice. However, the sensitivity of qPCR targeting minicircle kDNA can vary in function of species, complex or subgenus (depending on minicircle classes composition). Therefore, dependent on species in a particular geographical area, different qPCR assays to detect different taxa with high sensitivity can be run in parallel or sequentially to obtain an indication of subgenus or complex (through melting analysis or relative abundance of target sequence), as well as quantitative information. Then, based on results obtained, other qPCR assays can be selected for genotyping at the species level. The use of SYBR green coupled to melting or HRM analysis will help to reduce cost but will not allow multiplexing. Moreover, in cases of clinical samples with low parasite content, a nested approach can be used to enrich the target sequence before the application of species-specific qPCR assays, therefore avoiding late amplification (Ct ˃ 30) or failure to reach a plateau, in order to limit inconclusive or low-reproducible results in HRM analysis [105]. Despite considerable progress made in recent years, real-time PCR is still far from the clinical routine application in endemic areas. The cost of equipment and reagents is an important factor that hinders the transition from research to routine clinical application in endemic areas. However, miniaturization of real-time PCR equipment, as well as increased affordability of qPCR reagents could increase the routine use of the qPCR approaches in low-income countries.

## Abbreviations

6PGD: 6-phosphogluconate dehydrogenase
CL: Cutaneous leishmaniasis
Ct: Threshold cycle
FRET: Fluorescence resonance energy transfer
G6PD: Glucose-6-phosphate dehydrogenase
GPI: Glucose phosphate isomerase
HRM: High resolution melt
HSP70: Heat-shock protein 70
kDNA: Kinetoplast DNA
MCL: Mucocutaneous leishmaniasis
MPI: Mannose phosphate isomerase
qPCR: Quantitative real-time PCR
VL: Visceral leishmaniasis
